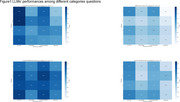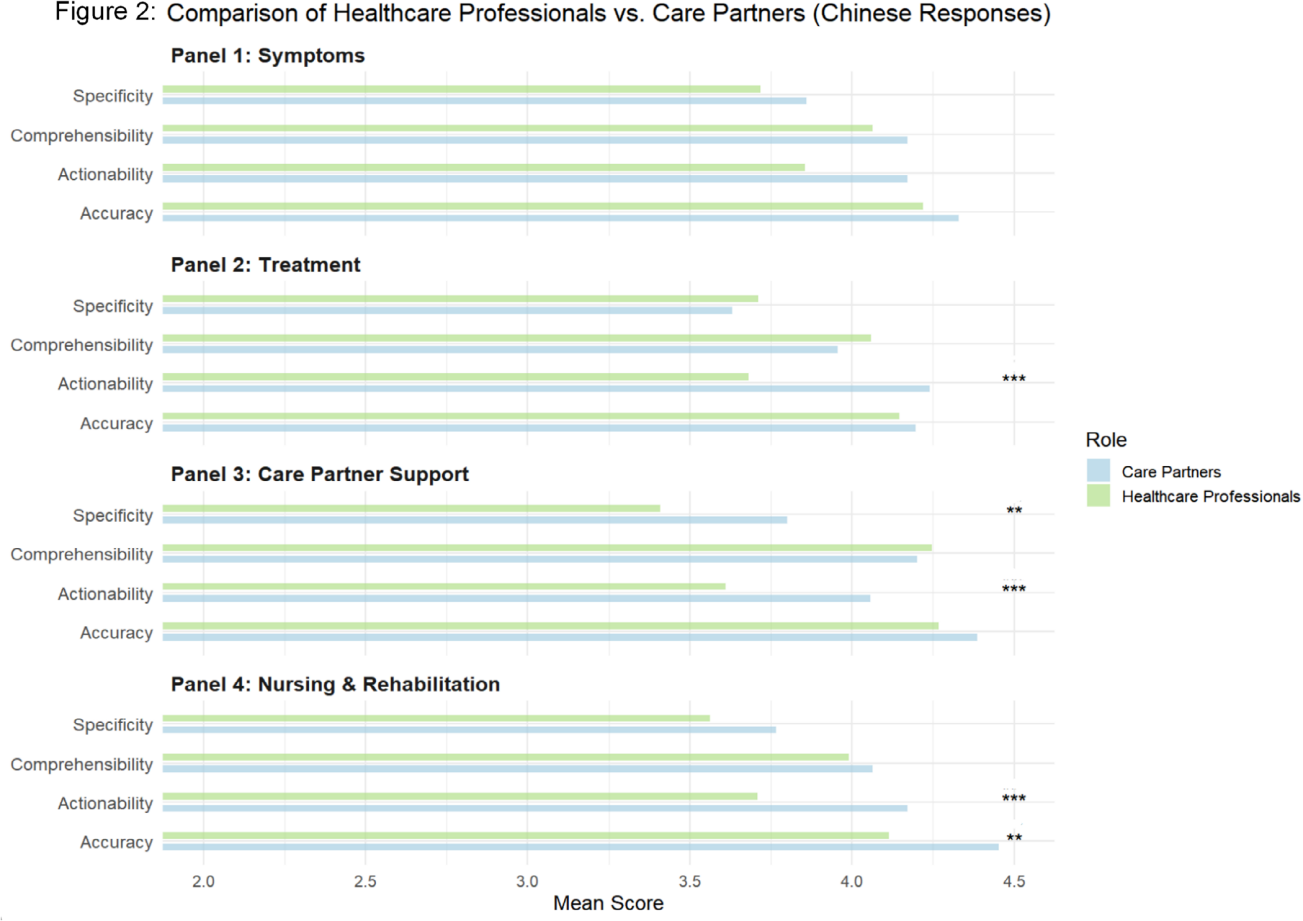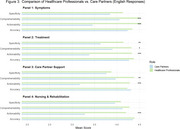# Assessing the role of Large Language Models in Mild Cognitive Impairment Management: A Bilingual Analysis Comparing ChatGPT, Gemini, and Kimi

**DOI:** 10.1002/alz70858_105517

**Published:** 2025-12-26

**Authors:** Yexuan Xiao, Qianhui Pan, Nan Jiang

**Affiliations:** ^1^ Tsinghua University School of Healthcare Management, Beijing, Beijing, China

## Abstract

**Background:**

The demand for accessible and actionable information to support mild cognitive impairment (MCI) management is growing, particularly for care partners and non‐psychiatric healthcare professionals seeking reliable guidance. Large Language Models (LLMs) have shown potential across various domains but have not been systematically evaluated in addressing MCI‐related queries. A critical gap exists in understanding LLM performance across different aspects of dementia care and the impact of language differences on their effectiveness. This study addresses these gaps by assessing LLM‐generated responses in both English and Chinese, providing essential insights into their capabilities and limitations.

**Method:**

Healthcare professionals (*N* = 5) and care partners (*N* = 5) were recruited from diverse clinical and caregiving backgrounds. A set of 72 open‐ended questions across four MCI domains—Symptoms, Treatment, Care partner support, and Nursing&rehabilitation—was developed. Responses from three LLMs (ChatGPT‐4, Gemini, Kimi) were evaluated by a five‐point accuracy scale using a double‐blind design. Ratings were based on accuracy, comprehensibility, specificity, and actionability. Statistical analyses included ICCs for reliability and Mann‐Whitney U tests to compare responses.

**Result:**

Among the four domains of MCI management, ChatGPT had the best performance in symptoms‐related queries (average score: 4.03, all *p* < 0.001). All three LLM‐generated responses demonstrated better alignment with healthcare professionals' requirements, achieving significantly higher concordance in comprehensibility (HP:4.24 vs CP:4.06, *p* <0.001) and actionability (HP:4.04 vs CP:3.72, *p* <0.001). Performance parity was observed in accuracy (CP:4.32 vs HP:4.27) and specificity (CP:3.79 vs HP:3.83) with no statistical significance. Additionally, English responses surpassed Chinese responses in accuracy (4.32 vs. 4.26, *p* = 0.056), comprehensibility (4.23 vs. 4.10, *p* < 0.001), and specificity (3.96 vs. 3.66, *p* < 0.001), while actionability scores showed no significant difference (3.94 vs. 3.89, *p* = 0.239).

**Conclusion:**

This study demonstrates LLMs' proficiency in symptom‐related inquiries and stronger alignment with healthcare professionals' operational needs versus care partners' accuracy priorities. English responses outperformed Chinese due to corpus disparities, highlighting needs for language‐specific optimization. Findings underscore LLMs' clinical potential, urging enriched Chinese medical corpora and specialized model development to address care partners' unmet requirements.